# Determination of the α‑Tocopherol Content in Edible Seed Oils *via* HPLC and an Electrochemical Approach

**DOI:** 10.1021/acsomega.6c03350

**Published:** 2026-07-22

**Authors:** Tugca Bilenler Koc, Ebru Kuyumcu Savan, Dilek Kazıcı, Ülkühan Bağış, Ihsan Karabulut

**Affiliations:** † Department of Food Engineering, Faculty of Engineering, 37520 Inonu University, Malatya 44280, Turkey; ‡ Division of Analytical Chemistry, Department of Basic Pharmaceutical Sciences, Faculty of Pharmacy, Inonu University, Malatya 44280, Turkey; § Zara Ahmet Çuhadaroğlu Vocational School, Food Processing, Food Quality Control and Analysis Program, Cumhuriyet University, Sivas 58700, Turkey

## Abstract

α-Tocopherol (α-T) is a fat-soluble antioxidant known as vitamin E. Vitamin E exists in eight different forms. Nevertheless, α-T is the most active and biologically effective of these forms. It protects cell membranes, fat tissues, and other bodily structures from oxidative damage by combating free radicals within the body. In this study, the α-T contents of two different edible seed oils (wheat germ and pomegranate seed) were compared *via* innovative (electrochemical) and traditional (high-performance liquid chromatography, HPLC) techniques to determine the consistency between the methods. The electrochemical determination of α-T in vegetable oils was performed *via* differential pulse voltammetry (DPV) at a multiwall carbon nanotube-modified glassy carbon electrode in a sodium perchlorate–acetonitrile medium. Additionally, the cyclic voltammogram of α-T showed two well-defined oxidation peaks. Using DPV, it was observed that the peak currents increased linearly with increasing α-T concentration in the range of 15.9–182.5 μg/L, with a detection limit of 0.23 μg/L (0.54 nM). The developed electroanalytical method was applied to detect α-T amounts in vegetable oils, and the results were in agreement with those obtained *via* HPLC.

## Graphical abstract

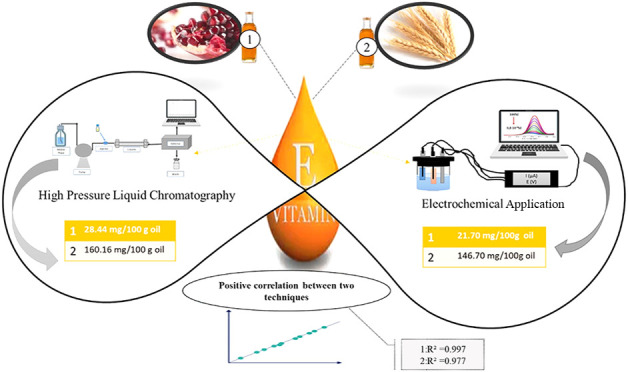

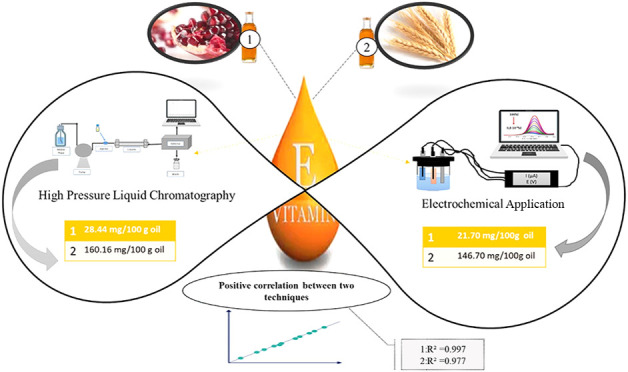

## 1 Introduction

Oxidative stability, a significant indicator of the oil quality and shelf life of edible seed oils, is affected by vitamin E isomers such as tocopherols.^1^ Tocopherols, phenolic and fat-soluble antioxidants, are naturally found in vegetable oils, eggs, fish, and the liver.^2^ Tocopherols act as radical scavengers by donating a hydrogen atom to the peroxyl radical of an unsaturated molecule and forming hydroperoxide. As a result of this reaction, lipid oxidation is delayed and functional products are protected against oxidative degradation. More stable, nonradical products are formed if another antioxidant reduces tocopherol radicals.^3^

There are four tocopherol isomers, namely, α, β, γ, and δ, depending on the degree of methylation of the aromatic phenolic ring of the chroman-6-ol structure.^4^ Among the four isoforms, fully methylated α-T is the most commonly used and biologically active tocopherol.^2,4^ The ability of a-T to prevent and treat chronic and degenerative diseases such as cardiovascular diseases, cancer, Alzheimer’s disease, and Parkinson’s disease has been well documented. Therefore, it is crucial to determine the amount of α-T found in foods to obtain the optimal level of intake in daily diets.^5^

Experimental studies on tocopherols began in the 1930s. Synthetic tocopherol production was achieved after isolation in the 1930s, purification in 1935, and structural elucidation in 1938.^4^ The tocopherol contents in foods, however, have been measured *via* a variety of methods, such as spectrophotometry,^6^ (HPLC)^7^ liquid chromatography-mass spectrometry (LC–MS),^8^ gas chromatography-supercritical liquid chromatography (GC–SLC),^9^ and ultraperformance liquid chromatography coupled with quadrupole time-of-flight mass spectrometry.^1^ Despite their advantages, these techniques also have several disadvantages. For example, in HPLC and GC techniques, although several HPLC-based methods, including those employing fluorescence detection (HPLC-FD), offer high sensitivity and selectivity without requiring derivatization steps, chromatographic techniques generally involve the use of toxic organic solvents and significant time consumption.^10^ Additionally, other disadvantages of these techniques are the low sensitivity and selectivity of the detector.^9,11^ Considering the disadvantages of currently used methods and the value of α-T, innovative analytical methods are highly important.

Electroanalytical methods are beneficial and widely used in the determination of organic compounds because of their sensitivity, selectivity, and accuracy and the important information they provide about electrochemical mechanisms similar to those occurring in metabolic processes. Some analytes can be determined down to nanomolar levels with different voltammetric techniques, which are used for electroanalytical analysis. α-T molecules have a phenolic hydroxyl group that can react with oxygen, so all these tocopherols can be electrochemically oxidized. These electrochemical properties provide new potentials and possibilities for the electroanalysis of α-T in vegetable oils and other products. Therefore, the electroanalytical method is considered an alternative to standard HPLC for determining the tocopherol content.^12^ In recent years, electrochemical sensors have attracted considerable attention for the determination of antioxidants and bioactive compounds because of their simplicity, rapid response, high sensitivity, and low operational cost.^13,14^ Various electrochemical approaches have been reported for tocopherol determination using different electrode materials, including glassy carbon electrodes, glassy carbon paste electrodes, and platinum electrodes in pure organic solvents or in their aqueous mixtures.^15^ Carbon nanotubes are particularly attractive electrode modifiers owing to their excellent electrical conductivity, high surface-to-volume ratio, electrocatalytic activity, and fast electron-transfer capability.^16^ Previous studies demonstrated that carbon nanotube-modified electrodes significantly enhance the electrochemical oxidation signal of α-tocopherol and improve analytical sensitivity.^12,17^ Differential pulse voltammetry is also widely preferred because of its superior sensitivity and minimized capacitive background current compared with conventional voltammetric techniques.^13^ For example, Ensafi et al.^16^ demonstrated the successful application of nanostructured electrode surfaces for sensitive electrochemical detection of bioactive compounds. Despite these advances, most reported studies focused primarily on standard solutions, pharmaceutical preparations, or synthetic systems rather than complex edible oil matrices.^15^ Moreover, studies simultaneously comparing electrochemical determination with conventional chromatographic techniques for edible seed oils remain very limited. Therefore, the development of reliable, sensitive, and cost-effective electrochemical platforms for α-tocopherol determination in edible oils continues to be of significant scientific and practical interest.

In this study, the voltammetric behavior of α-T at a modified glassy carbon electrode (GCE) based on multiwalled carbon nanotubes (MWCNTs) was investigated via cyclic voltammetry (CV), and DPV was used to develop a rapid and sensitive method for the quantitative determination of α-T in vegetable oils based on its electrochemical oxidation. The optimum experimental conditions of this electroanalytical method were determined, and the results of its application to various vegetable oils were validated by comparison with those obtained *via* HPLC.

Vegetable oils, which positively affect human health, are rich in phytonutrients (tocopherols, anthocyanins, and antioxidants).^18^ Determining the α-T value of vegetable oils is very important in terms of the functional and nutritional value of the oils.^19^ Wheat germ and pomegranate seed oils, which are the subject of this study, are considered important oils among edible-grade seed oils. Therefore, the objective of this study was to determine the α-T content in edible seed oils (wheat germ and pomegranate seed) *via* both traditional instrumental (HPLC) and innovative analytical (electrochemistry) techniques and to evaluate the consistency between the two methods. The novelty of the present study lies in the combined application of a multiwalled carbon nanotube-modified glassy carbon electrode (MWCNT/GCE) and differential pulse voltammetry for the sensitive determination of α-tocopherol directly in edible seed oil matrices, together with comprehensive validation against the conventional HPLC method. Although electrochemical approaches for tocopherol analysis have previously been reported, studies focusing on wheat germ oil and pomegranate seed oil and directly comparing electrochemical and chromatographic techniques are still scarce. The proposed electrochemical platform offers several advantages, including rapid analysis, minimal sample preparation, low solvent consumption, cost-effectiveness, and excellent analytical sensitivity with a nanomolar detection limit. Therefore, this work provides a practical and reliable alternative analytical strategy for routine α-tocopherol determination in edible oils.

## 2 Materials and Methodology

### 2.1 Samples, Reagents, and Standards

Two cold-pressed vegetable oils, wheat germ (*Triticum sativum*) and pomegranate (*Punica granatum* L.) seed oils, were purchased from a local market, and the oil samples were stored at 4 °C until further analyses. All analyses were performed in triplicate. Hexane, isopropanol, tetrabutylammonium tetrafluoroborate (TBATFB), LiClO_4_, NaClO_4_, ethanol, (EtOH), acetonitrile (ACN), and dimethylformamide (DMF) were obtained from Merck (Darmstadt, Germany). α-Tocopherol was purchased from Sigma Aldrich (St. Louis, MO). All reagents were of analytical purity. MWCNTs (average diameter: 3–10 nm, length: 10–30 μm, purity: >95%, surface area: 500 m^2^/g) were obtained from Graphene (Ankara, Turkey). The supporting electrolyte solutions with a concentration of 0.1 M used in the voltammetric measurements were prepared in EtOH and ACN just before analysis. Before performing the voltammetric study, all the solutions were exposed to nitrogen gas for 2 min.

### 2.2 α-Tocopherol Analysis

#### 2.2.1 Chromatographic Measurement

A Shimadzu HPLC system integrated with an autosampler (SIL-20A HT) including a column oven (CTO-10AS VP), a degasser system (DGU2A5R), a gradient pump (LC-20AR), a diode-array detector (DAD, SPDM20A), and a software package for system control and data acquisition (LC solution) was used for analyses. Oil samples were dissolved in hexane at a concentration of 10 mg/mL (1 g of oil per 100 mL of hexane) and filtered through a 0.45 μm nylon filter (Lubitech, Songjiang, China); 20 μL of the solution was injected into the column, and analysis was performed according to the methods of Karabulut et al.^20^ Separations were achieved on an inertial NH_2_ column (250 mm × 4.6 mm, 5 mm; GL Sciences Inc., Tokyo, Japan) at 30 °C *via* isocratic elution. The mobile phase used was *n*-hexane (99%) and isopropanol (1%), and the flow rate was 1 mL/min. Detection was carried out at 292 nm. Quantification was carried out *via* the use of a calibration curve prepared with 7-point concentrations of the NH_2_ column α-T standard. The concentrations were expressed as mg/100 g of oil.

#### 2.2.2 HPLC Method Validation

The HPLC analytical method was validated in-house in accordance with the procedures specified in the International Conference on Harmonisation (ICH) (2005) guideline.^21^ The method performance was confirmed based on the following criteria: selectivity, linearity, accuracy (recovery), precision, limit of detection (LOD), and limit of quantitation (LOQ). Selectivity was confirmed by the absence of coeluting matrix peaks at the retention time of α-T in both oil matrices (Figure S1). Linearity was assessed using a seven-point calibration curve prepared from α-T standard solutions in n-hexane at concentrations of 1.95–125 μg/mL. Each solution was injected in triplicate, and the mean peak area was plotted against concentration. Accuracy was determined by calculation of the recovery rates. For this purpose, α-T was spiked at three different concentrations (low, medium, and high) to both oil samples, which had known α-T concentrations. Wheat germ oil (spiked with 50, 100, and 200 mg of α-T/100 g of oil) and pomegranate seed oil (spiked with 10, 25, and 50 mg of α-T/100 g of oil) samples were analyzed in triplicate at each level (*n* = 9). Precision was evaluated based on the relative standard deviation (RSD%) obtained from replicated analysis of spiked samples at given concentrations. The LOD and LOQ were obtained from the oil samples diluted in n-hexane to a final α-T concentration of 2 μg/g oil. The LOD and LOQ were determined as 3- and 10-fold of the standard deviation calculated from 10 replicated analyses of diluted samples, respectively. All validation parameters are summarized in Table 1.

**Table 1:** Validation Parameters for Both HPLC and Electrochemical Approaches

parameter	electrochemical (DPV, MWCNT/GCE)	HPLC (UV, 292 nm, NH_2_ column)
calibration equation	*I*(μA) = 0.6157*C*(μg/L) – 6.7935	*y =* 0.00012351*x* + 1.24286
detection mode	715 (mV)	292 nm (UV)
linear range	15.9–182.5 (μg/L)	1.95–125 μg/mL
slope	0.6157 (μA·μg/L)	0.00012351(AU.g/μg)
intercept	–6.7935 (μA)	1.24286 (AU)
correlation coefficient (*R* ^2^)	0.9901	0.9990
recovery(%) low spike		92.12 ± 2.13 (WGO), 88.26 ± 2.21(PO)
recovery(%) medium spike		97.49 ± 1.13 (WGO), 91.57 ± 1.14 (PO)
recovery(%) high spike		106.32 ± 2.01 (WGO), 107.48 ± 1.12 (PO)
LOD	0.231 (μg/L)	1.54 (μg/g)
LOQ	0.769 (μg/L)	5.13 (μg/g)

#### 2.2.3 Electrochemical Measurement

Electrochemical measurements were performed *via* CV and DPV methods on a Gamry Interface 1010B (Gamry, USA) potentiostat and triple-electrode system (BASi C3 Cell Stand). A GCE was used as the working electrode, saturated Ag/AgCl was used as the reference electrode, and Pt wire was used as the counter electrode. The GCE surface was cleaned on cleaning pads with 0.3 and 0.05 μm Al_2_O_3_ and then washed with ultrapure water obtained from a Milli-Q system (Millipore, 18.2 MΩ·cm, Merck). Additionally, the GCE was electrochemically cleaned *via* the CV method in 0.5 M H_2_SO_4_ solution for 10 cycles between −0.5 and 2.0 V at a scan rate of 100 mV. The required reagents were weighed on a Weightlab Instruments/WSA-224 precision balance. The MWCNTs used for the modification of the GCE surface were prepared by sonicating in DMF with an ultrasonic bath (DK Sonic) for 4 h at a concentration of 0.5%. The mixture was vortexed with a vortex mixer (Wiggens/Vortex 3000) before being dropped onto the electrode surface. The modified sensor (MWCNT/GCE) was subsequently obtained by dropping this mixture onto the GCE surface and drying it at room temperature.

Before electrochemical measurements, a minimum of 100 μL of oil sample was used per measurement; samples were diluted 1:10 (v/v) with 0.1 M NaClO_4_ prepared in acetonitrile (ACN). The mixture was vortexed thoroughly to obtain a homogeneous solution prior to voltammetric analysis. Since the electrochemical measurements were carried out in a nonaqueous ACN-based electrolyte medium, no aqueous phase or emulsion formation occurred during the analysis.

## 3 Results and Discussion

This study presents a comprehensive cross-validation of HPLC and electrochemical approaches to determine the α-T content of vegetable oils and evaluate the agreement between the two methods. HPLC analysis revealed the presence of α-T in the two tested vegetable oils (Figure S1).

The HPLC method was validated prior to sample analysis and demonstrated sufficient analytical performance for all assessed parameters (Table 1). A calibration curve was established in the range of 1.95–125 μg/mL, and this curve exhibited sufficient linearity (*R* ^2^ ≥ 0.9990) for the quantitative determination of α-T within the concentration range studied. Mean recovery values of 92.12 ± 2.13% (low), 97.49 ± 1.13% (medium), and 106.32 ± 2.01% (high) for wheat germ oil (WGO) and 88.26 ± 2.21% (low), 91.57 ± 1.14% (medium), and 107.48 ± 1.12% (high) for pomegranate seed oil (PO) were obtained. The relative standard deviation (RSD%) obtained from recovery trials ranged from 1.15% to 2.31% for WGO and from 1.04% to 2.50% for PO across all spiking levels, indicating satisfactory repeatability of the overall analytical procedure. The LOD and LOQ values were determined to be 1.54 μg/g and 5.13 μg/g, respectively. These values demonstrated that the HPLC method is sufficiently suitable for detecting even low concentrations of α-T in the oils.

According to the validated HPLC analysis results, wheat germ oil contained 160.16 mg/100 g α-T and pomegranate seed oil contained 28.44 mg/100 g α-T. The amount of α-T determined in the wheat germ oil in this study was much greater than the value (80.27 mg/100 g) determined by Kommoun et al.,^22^ which is consistent with other literature results.^23,24^ Calligani et al. reported that the amount of α-T in pomegranate seed oil varies between 13.5 and 42.3 mg/100 g of oil, which is consistent with our findings.^25^ The HPLC results served as reference values for cross-validation with the proposed electrochemical method (Table 2).

**Table 2:** α-Tocopherol Content in Oils Determined by HPLC and Electrochemical Techniques (mg/100 g of Oil)^a^

samples	HPLC		electrochemical		*R* ^2^
wheat germ	160.16	±0.01	146.70	±0.01	0.977
pomegranate seed	28.44	±0.02	21.70	±0.00	0.997

a Values are expressed as mean ± standard deviation (*n* = 3). R2^2^ represents the Pearson correlation coefficient between HPLC and electrochemical results.

To perform quantitative electrochemical determination of α-T, the solution medium in which the analysis is performed (supporting electrolyte solution), the film thickness of the designed sensor surface, and the parameters of the voltammetric technique must be optimized. Using a supporting electrolyte solution is critical for the accuracy, repeatability, and sensitivity of electrochemical analyses. The use of electrolytes provides a stable potential distribution in the electrochemical cell, minimizes side reactions, and increases the reliability of the analysis results. Therefore, to determine the supporting electrolyte during electrochemical studies, the DPV responses of the MWCNT/GCE-modified sensor were investigated for 0.6 mg/L α-T in solutions of LiClO_4_, NaClO_4_, and TBATFB in EtOH and LiClO_4_, NaClO_4_, and TBATFB in ACN with concentrations of 0.1 M (Figure 1). The electrolyte medium in which the best peak current for α-T was obtained was determined to be NaClO_4_ dissolved in ACN, and this electrolyte was used in all subsequent analyses.

**Figure 1:**
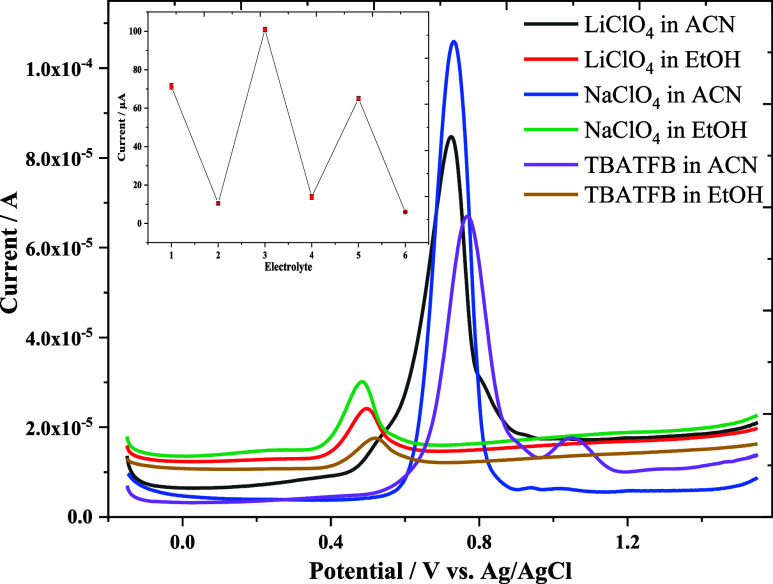
DPV responses of 0.6 mg/L α-T at the MWCNT/GCE-modified sensor in different supporting electrolyte solutions.

Film thickness is an important parameter in the modification of the electrode surface in electrochemical analysis, and this thickness directly affects the performance and sensitivity of the electrode. For this reason, the film thickness at which the highest peak current response could be obtained for the electrochemical oxidation of α-T was investigated. MCNTs (0.5% MCNTs) were dropped onto the GCE surface separately and with different film thicknesses (2, 4, 5, 6, 8, and 10 μL), and 1 mg/L α-T was determined in 0.1 M NaClO_4_ *via* the DPV technique at these sensors (Figure S2). The best peak current for α-T was measured with the sensor with a film thickness of 6 μL, and the modified sensor with this thickness was used in all subsequent analyses.

In voltammetric methods, signal responses vary with the parameters of the device. For this reason, the pulse size, sample period, and step size parameters were optimized for the DPV method used in the electrochemical analyses in this study (Figure S3). Pulse size optimization was performed with values of 10, 25, 50, 75, and 100 mV (Figure S3a). As the pulse size increased, the peak potential shifted to positive values and the peak current increased. However, since reproducible results could not be obtained with very small and very large pulse sizes, the pulse size was determined to be 50 mV. Sample period optimization was performed with values of 0.2, 0.4, 0.6, 0.8, and 1.0 s (Figure S3b). As a result of the voltammetric measurements, the best peak current was obtained at 0.2 s, and this value was used in the subsequent electrochemical analyses. Step size optimization was performed with values of 1, 2, 3, 4, and 5 mV (Figure S3c). The best peak current was obtained at 1 mV, and this value was used in the subsequent electrochemical analyses.

To study the electrochemical behavior of α-T, measurements were carried out at increasing scan rates (10, 25, 50, 75, 100, 125, 150, 175, and 200 mV/s) *via* the CV method in an ACN solution containing 0.1 M NaClO_4_ under optimum conditions (Figure 2). Two anodic oxidation peaks were observed at approximately 0.5 and 0.75 V, and two cathodic reduction peaks were observed at 0.3 and 0.7 V. As the scanning rate increased, the peak current increased, the anodic peak potential shifted to positive, and the cathodic peak potential shifted to negative.

**Figure 2:**
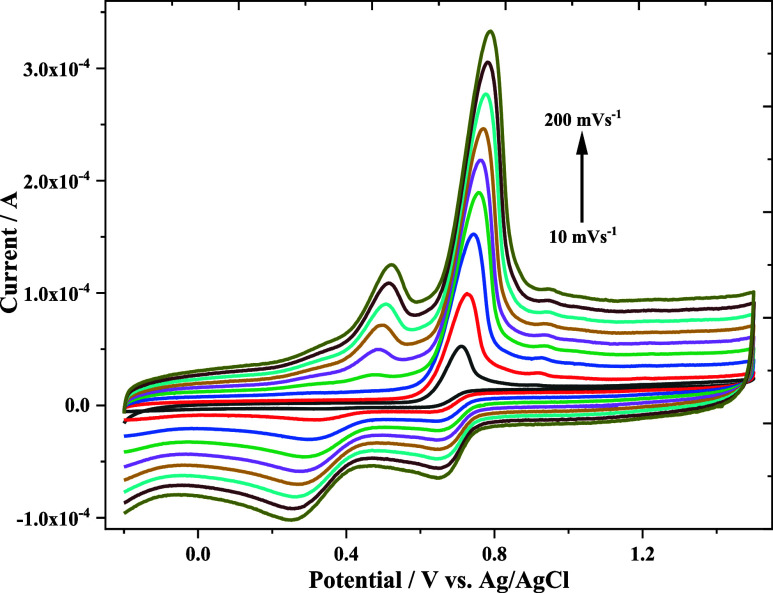
CV responses at different scanning rates at the MWCNT/GCE-modified sensor for 1 mg/L α-T.

To determine whether the transport of α-T to the MWCNT/GCE-modified sensor surface was diffusion- or adsorption-controlled, graphs of increasing scan rates versus peak currents were drawn, and a linear relationship was detected (Figure S4a). In addition, peak currents were plotted against the square root of the scan rate, and a linear relationship was detected (Figure S4b). When both graphs are examined, it is thought that α-T may be adsorbed on the MWCNT/GCE-modified sensor surface. To verify this information, the linear relationship between the logarithm of the oxidation peak current and the logarithm of the scan rate was examined (Figure S4c). Theoretically, if the slope value of the linear curve obtained from the log*Ip*–log υ graph is close to 0.5, it is considered diffusion-controlled, and if the slope value of the linear curve is close to 1.0, it is considered adsorption-controlled. When Figure S4c was examined, the slope of the linear curve obtained from the log*Ip*–log υ graph was determined to be 0.6014, and since it is close to the theoretical value of 0.50, it confirms the diffusion-controlled mechanism.

Laviron’s equation was used to obtain information about the electrons involved in the oxidation of AMH on the biosensor surface.^26^ According to the Laviron equation, the number of electrons was calculated from the slope of the line equation obtained by plotting *E_p_* versus log *v* (Figure S4d). Based on these data, the number of electrons was calculated as 1.9 (≈2); thus, two electrons play a role in the oxidation of α-T on the MWCNT/GCE-modified sensor surface.

A calibration chart was created from the current data measured against increasing concentrations of α-T at the MWCNT/GCE-modified sensor under optimum conditions (Figure 3). The limit of detection (LOD) and limit of quantitation (LOQ) values of α-T were calculated from the calibration chart data. The LOD is 3 s/m, and the LOQ is 10 s/m. “*m*” is the slope of the calibration graph drawn against the oxidation peak currents of increasing concentrations of α-T. “*s*” is the standard deviation of the voltammetric oxidation peak currents obtained by measuring α-T at the lowest concentration 10 times.

**Figure 3:**
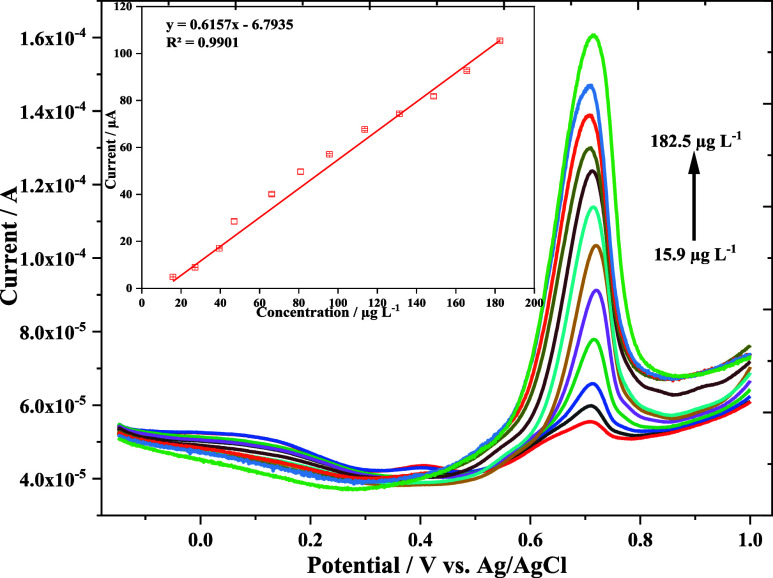
DPV responses of increasing concentrations of α-T in 0.1 M NaClO_4_ at the MWCNT/GCE-modified sensor. Inset: calibration curve of α-T in the linear range of 15.9–182.5 μg/L.

Increasing concentrations of α-T, ranging from 15.9 to 182.5 μg/L in 0.1 M NaClO_4_ solution, were measured at the MWCNT/GCE-modified sensor *via* the DPV method. As the concentration of α-T increased, the peak potentials also increased (Figure 3). The regression equation of the calibration graph was recorded as *I*(μA) = 0.6157*C*(μg/L) – 6.7935 (*R* ^2^ = 9901). The LOD and LOQ values of α-T were 0.231 μg/L (0.54 nM) and 0.769 μg/L (1.79 nM), respectively. The validation parameters are summarized in Table 1. Additionally, the repeatability of the MWCNT/GCE-modified sensor was tested. 1.0 ppm α-T was measured at the same modified sensors by DPV (Figure S5), and relative standard deviation (RSD) was calculated as 1.34%. 10 different modified sensors were prepared to determine the reproducibility of the proposed modified sensor. 1.0 ppm α-T was measured at 10 different modified sensors by DPV (Figure S6). Very close peak current values were obtained, and RSD was calculated as 9.48%. These results prove that the manufactured modified sensor shows excellent stability and is highly sensitive even at very low concentrations.

Although the calculated detection limit was 0.231 μg/L, the validated linear working range was determined as 15.9–182.5 μg/L. Concentrations below 15.9 μg/L produced detectable electrochemical responses; however, these signals did not exhibit sufficient linearity and reproducibility for reliable quantitative evaluation. Therefore, concentrations lower than 15.9 μg/L were not included in the calibration range. Such differences between the statistically calculated LOD value and the experimentally validated linear range are commonly observed in electroanalytical studies.

The α-T contents of the oil samples were determined *via* DPV (Figure S7). All the samples were diluted 1:10 with 0.1 M NaClO_4_ solution. The concentrations of the oil samples were calculated from the measured current values of α-T and the calibration curve.

Electrochemical analysis revealed that α-T could be determined well in the tested vegetable oils. The α-T contents in pomegranate seeds and wheat germ were 21.70 mg/100 g of oil and 146.70 mg/100 g of oil, respectively. The agreement between the voltammetric technique and HPLC results was evaluated *via* regression analysis, and the Pearson correlation coefficient between the two methods is shown in Table 2. A strong correlation was observed between the HPLC and voltammetric values in both wheat germ oil (*R* ^2^ = 0.977, *p* < 0.01) and pomegranate seed oil (*R* ^2^ = 0.997, *p* < 0.01).

Although edible oils contain various naturally occurring compounds such as sterols, pigments, free fatty acids, and other tocopherol isomers, the optimized nonaqueous electrolyte medium provided a well-defined electrochemical response for α-T under the selected voltammetric conditions. In addition, the strong agreement between electrochemical and HPLC results suggests that possible matrix interferences did not significantly affect the α-T determination. According to previously reported compositional studies, α-T is one of the predominant tocopherol forms in wheat germ and pomegranate seed oils; therefore, the measured oxidation signal was mainly attributed to α-T oxidation.

The strong agreement between the electrochemical measurements and the reference HPLC method demonstrates the analytical accuracy and applicability of the proposed sensor platform for real edible oil samples. Since HPLC is a well-established and validated analytical technique for α-T determination, the high correlation between the two independent methods confirms that matrix interference effects were minimal under the optimized nonaqueous experimental conditions. Therefore, the comparative validation with HPLC was considered sufficient to verify the reliability of the proposed electrochemical method for practical applications.

As a result, the analytical parameters, detection methods, and LOD values for the electrochemical determination of α-T with various modified sensors in the literature are compared in Table 3. According to these results, the modified sensors detect α-T with very low LODs over a wide measurement range, the modified sensor has a straightforward sensor preparation method, and this study does not include a complex electrolyte system. In addition, the sample preparation step and detection time are shorter than those of many techniques, such as HPLC. Thus, a modified sensor that can be used practically for a long time, can make accurate, sensitive, and precise measurements, and has a very fast response has been prepared.

**Table 3:** Voltammetric Studies of α-Tocopherol

modified sensor	voltammetric technique	supporting electrolyte	LOD	linear range	refs	matrix
PPy/Pt	DPV	1,2-dichloroethane−ethanol	1.5 μM	5.0–300 μM	^12^	vegetable oil
GCE	DPV	0.02 M sulfuric acid–0.06 M TMAC in 40:60 hexane−ethanol	2.0 μM	0.46–1.16 μM	^27^	vegetable oil
GCE	SWV	0.02 M sulfuric acid–0.06 M TMAC in 40:60 hexane−ethanol	1.49 μM	0.46–1.16 μM	^27^	micellar media/buffer
Pt electrode	CV	Et_4_NOH in acetonitrile	-	0.5–10 mM	^28^	standard solution (ACN)
CPE	DPV	Triton X-100 + acetonitrile + water	0.6 μg/cm^3^	1.5–200 μg/cm^3^	^29^	surfactant/buffer solution
GCE	CV	PBS pH 7.4			^30^	standard solution (ACN)
poly(2,20-(1,4-phenylenedivinylene)-bis-8-hydroxyquinaldine)/MWCNT/GCE	SWV	Triton X-100	0.1 μM	8–100 μM	^31^	micellar media/buffer, vegetable oil
GCE	CV	0.1 M LiClO_4_ in ACN	1.02 μM	2–140 μM	^32^	standard solution (ACN)
Pt electrode	CV	0.2 M *n*-Bu_4_NPF_6_ in CH_3_CN		0.013–0.55 M	^33^	standard solution (ACN)
carbon fiber disk ultramicroelectrode	SWV	benzene/ethanol + 0.1 M H_2_SO_4_			^34^	micellar media/buffer
GCPE	SWASV	0.1 M HNO_3_	1 × 10^–7^ M, 3.3 × 10^–9^ M	5 × 10^–7^–4 × 10^–5^ M, 5 × 10^–8^–1 × 10^–5^ M	^35^	edible oil /standard
NF/ER-GO nanocomposite	SWV	acetone containing acetate buffer (0.1 M, pH 4)	0.06 μM	0.5–90 μM	^17^	micellar media/buffer
GCE	DPV	ethanol and water (70:30)		0.43–70.41 mg/dm^3^	^36^	real samples (oils, margarines)
MWCNT/GCE	DPV	0.1 M NaClO_4_ in ACN	0.54 nM	0.037–0.424 μM	this study	edible seed oil (wheat germ, pomegranate seed)

## 4 Conclusions

The α-T contents of wheat germ and pomegranate seed oils were evaluated via both traditional (chromatographic) and electrochemical techniques. While HPLC was used in chromatographic techniques, a sensor modified with an MWCNT/GCE was developed for electrochemical evaluation. The use of modified sensors and voltammetric techniques offers several advantages in terms of ease of use, fast results, cost-effective protocols, easy preparation of the modified sensor, and no lengthy sample preparation procedure. The electrochemical response of the modified sensor to α-T is linear at very low limits of detection. Good reproducibility and sufficient long-term stability make the MWCNT/GCE sensor a useful device. Chromatographic techniques and electrochemical approaches show good agreement in determining the α-T content in edible oils. The electrochemical detection approach is practically useful for the estimation of α-T in vegetable oils. This method offers an effective approach that can be used as a universal technique and a potential alternative for high-performance liquid chromatography. Moreover, the proposed detection method is faster and cheaper than the determination of α-T in vegetable oil samples by HPLC.

## Supplementary Material



## Notes

α-Tocopherol chromatograms for standard, wheat germ oil, and pomegranate seed oil, DPV responses at different film thicknesses of the MWCNT/GCE-modified sensor, DPV responses at different pulse sizes, sample periods, and step size values, CV responses at different scanning rates and corresponding calibration plots, repeatability and reproducibility of the MWCNT/GCE-modified sensor, and DPV responses for diluted oil samples (PDF)
