# Supplementary material for: Determination of the α‑Tocopherol Content in Edible Seed Oils via HPLC and an Electrochemical Approach
Source: ACS Omega. 2026 Jul 22;11(30):45069–76. doi: 10.1021/acsomega.6c03350 (PMC13448941; doi:10.1021/acsomega.6c03350)
Supplement: Supplementary file 1 [file ao6c03350_si_001.pdf]

Supporting Information

Determination of the alpha-tocopherol content in edible seed oils *via* HPLC and an electrochemical approach

Tugca Bilenler Koc[1*], Ebru Kuyumcu Savan[2], Dilek Kazıcı[2], Ülkühan Bağış[3] and Ihsan Karabulut[1]

[1] *Department of Food Engineering, Faculty of Engineering, Inonu University, Malatya, Turkey, 44280*

[2] *Division of Analytical Chemistry, Department of Basic Pharmaceutical Sciences, Faculty of Pharmacy, Inonu University, Malatya, Turkey, 44280*

[3] *Zara Ahmet Çuhadaroğlu Vocational School, Food Processing, Food Quality Control and Analysis Program, Cumhuriyet University, Sivas, Turkey, 58140*

*Corresponding author**:** tugca.bilenler@inonu.edu.tr

Contents

Fig. S1 Alpha tocopherol chromatograms. **a)** alpha tocopherol standard, **b)** wheat germ oil **c)** pomegranate seed oil………3

Fig. S2 DPV responses of 1.0 mg/L α-T at the different film thicknesses of MWCNT/GCE modified sensor….4

Fig. S3 DPV responses of 0.2 mg/L α-T in 0.1 M $NaClO_4$ at MWCNT/GCE modified sensor for **a)** different pulse size, **b)** different sample period, **c)** different step size values……………………………………..…5

Fig. S4 CV responses of 1 mg/L α-T at the MWCNT/GCE modified sensor at different scanning rates: **a)** *Ip* vs *v*, **b)** *Ip* vs $v^{1/2}$, **c)** log (*Ip* / μA) vs log (*v* / mV/s), and **d)** variation between *Ep* and log*v*……………………………….6

Fig. S5. Repeatability of the MWCNT/GCE modified sensor for 1.0 ppm α-T in 0.1 M $NaClO_4$ prepared in ACN………..7

Fig. S6. Reproducibility of MWCNT/GCE modified sensors for 1.0 ppm α-T in 0.1 M NaClO4 prepared in ACN………..8

Fig. S7. DPV responses at the MWCNT/GCE modified sensor as a result of diluting the oil samples with 0.1 M $NaClO_4$ in ACN solution………………………………………………………………………………………9

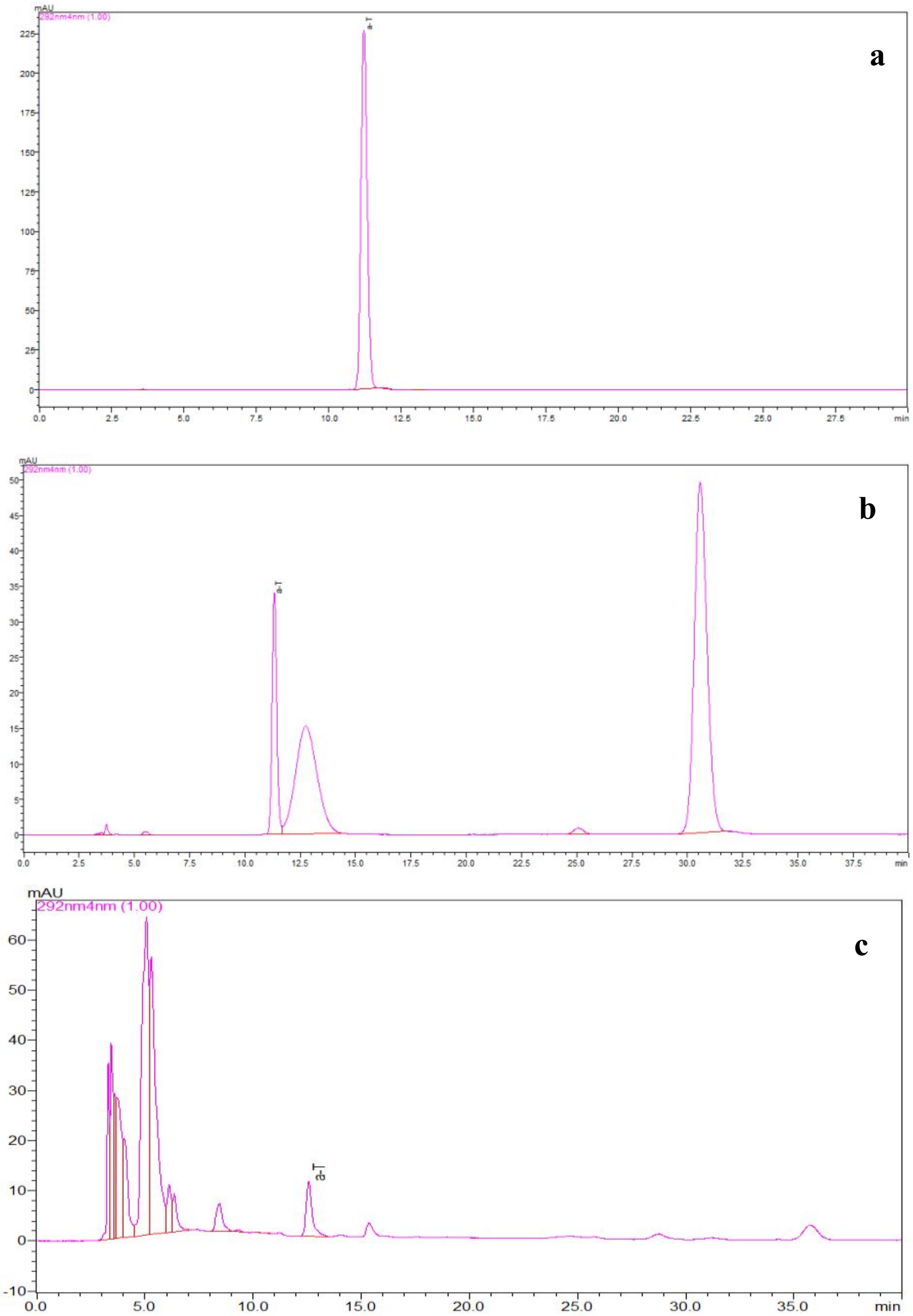

Fig. S1 Alpha tocopherol chromatograms. **a)** alpha tocopherol standard, **b)** wheat germ oil **c)** pomegranate seed oil

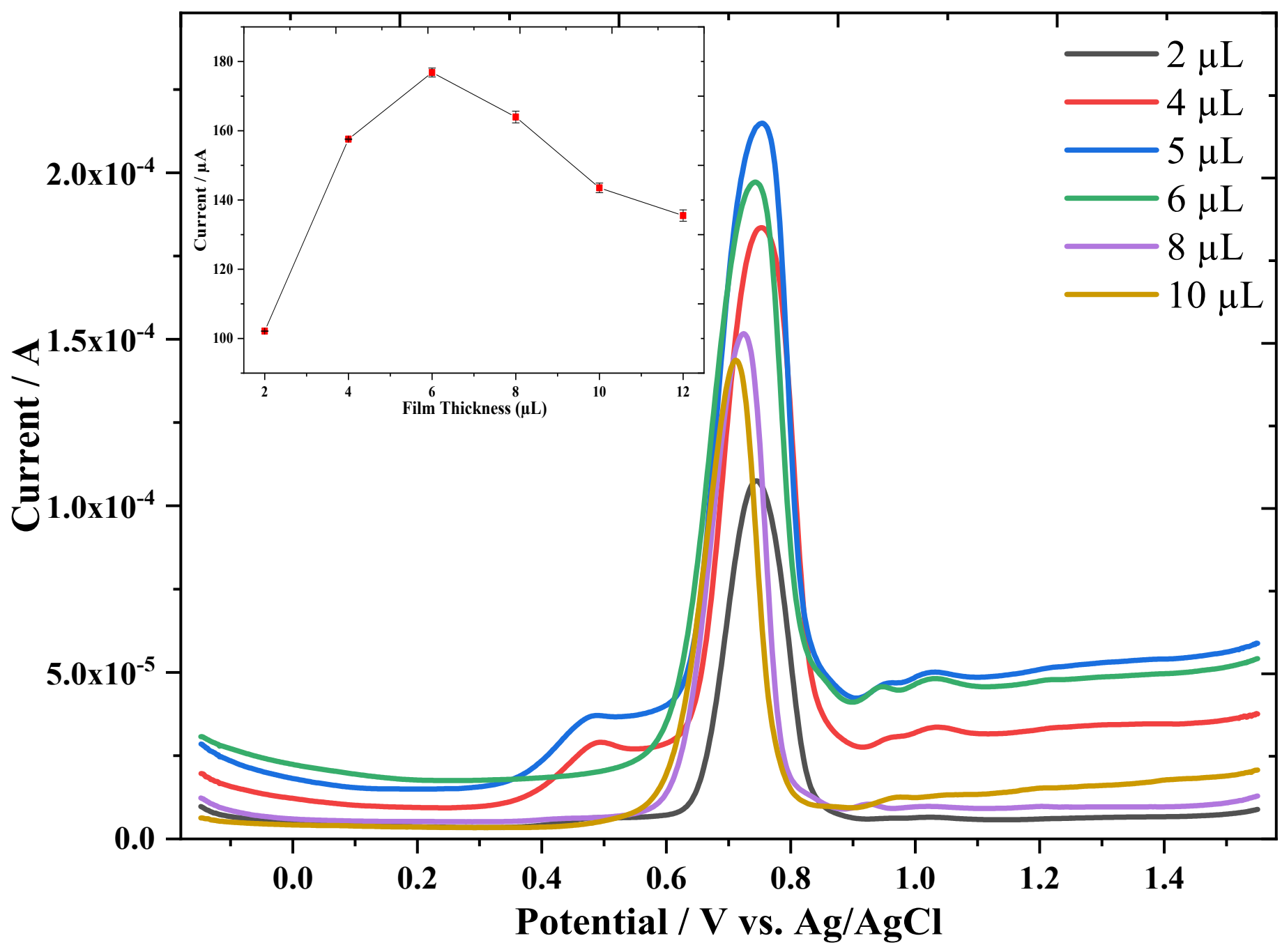

Fig. S2 DPV responses of 1.0 mg/L α-T at the different film thicknesses of MWCNT/GCE modified sensor.

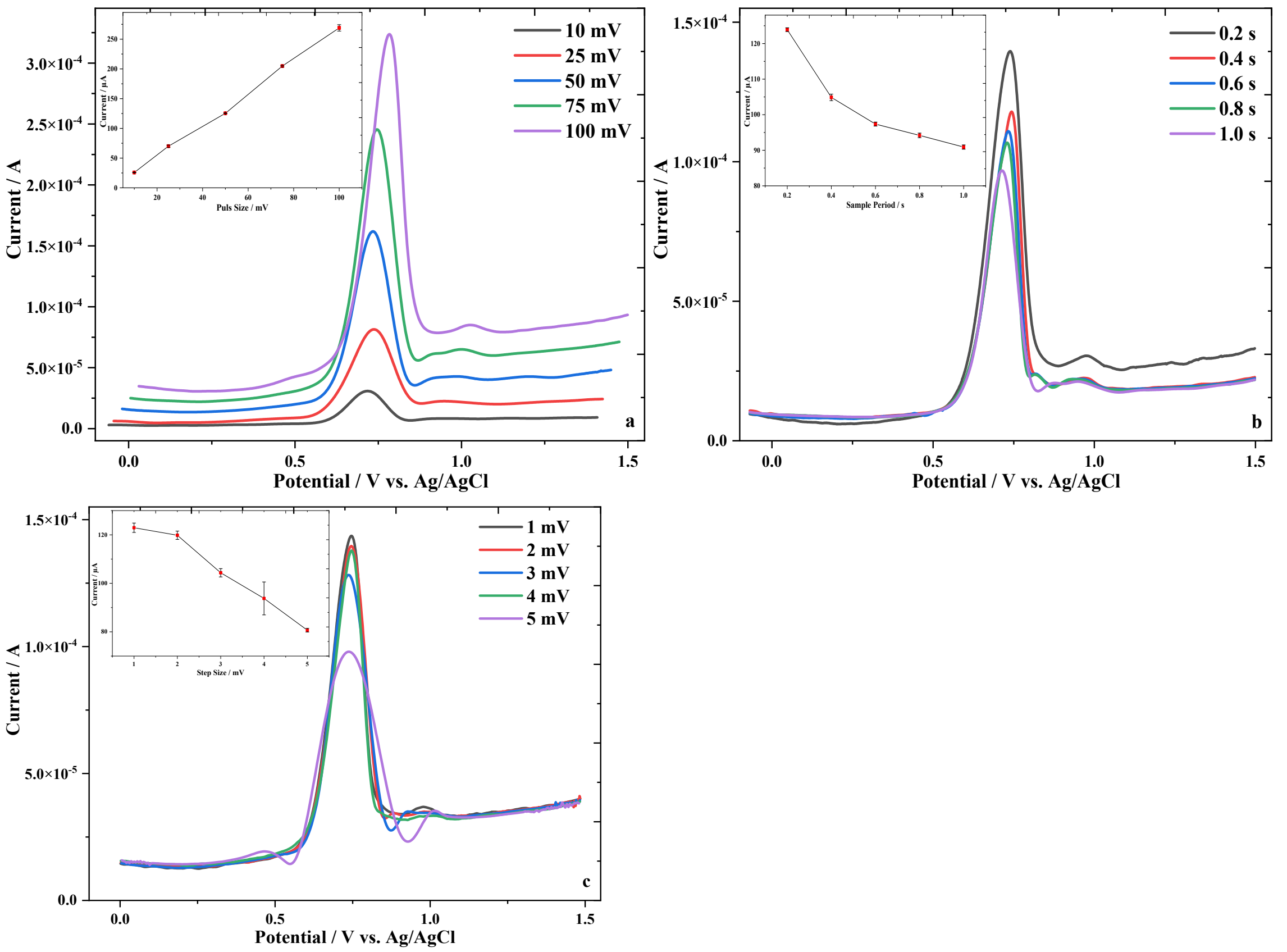

Fig. S3 DPV responses of 0.2 mg/L α-T in 0.1 M $NaClO_4$ at MWCNT/GCE modified sensor for **a)** different pulse size, **b)** different sample period, **c)** different step size values.

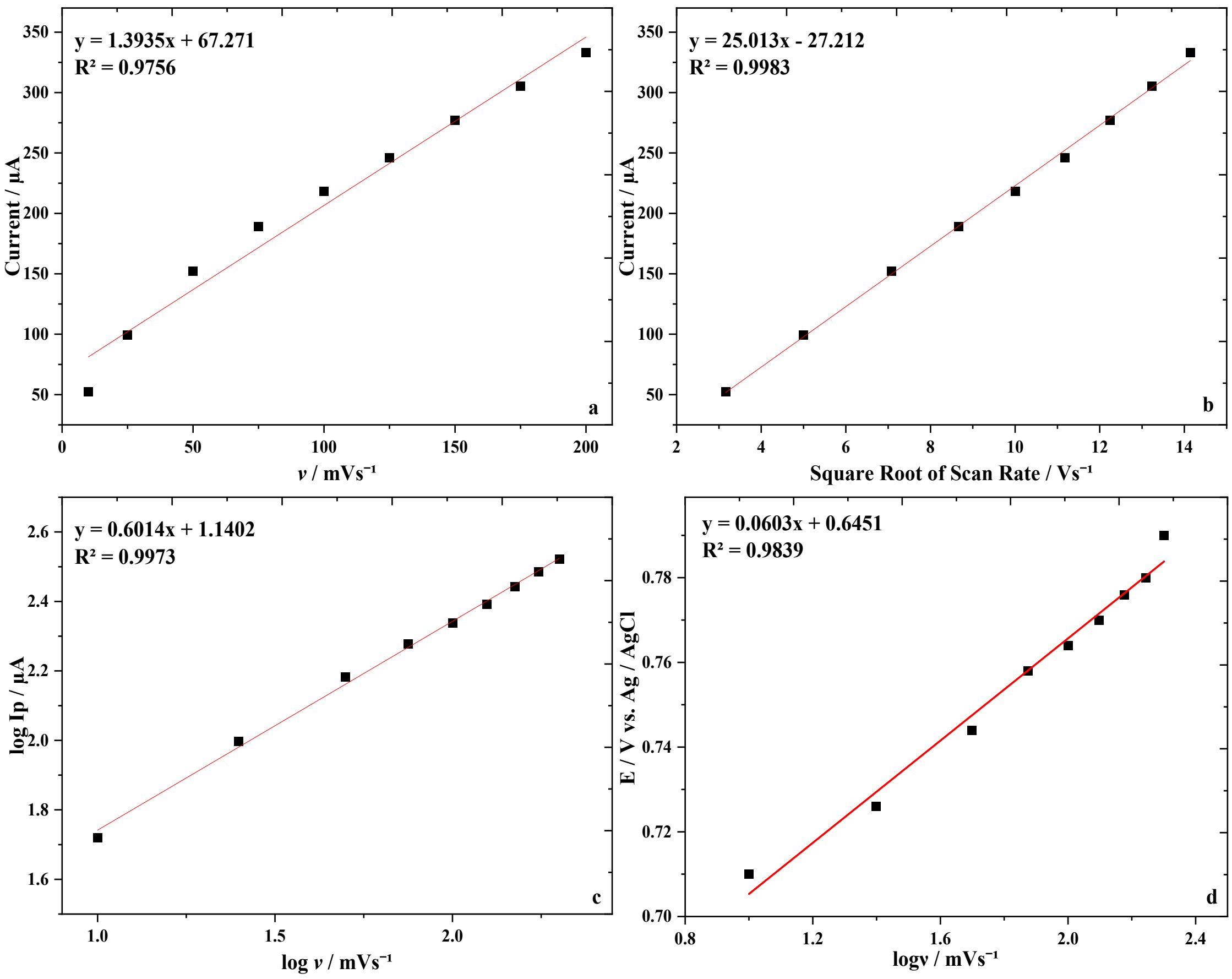

Fig. S4 CV responses of 1 mg/L α-T at the MWCNT/GCE modified sensor at different scanning rates: **a)** *Ip* vs *v*, **b)** *Ip* vs $v^{1/2}$, **c)** log (*Ip* / µA) vs log (*v* / mV/s), and **d)** variation between *Ep* and log*v*.

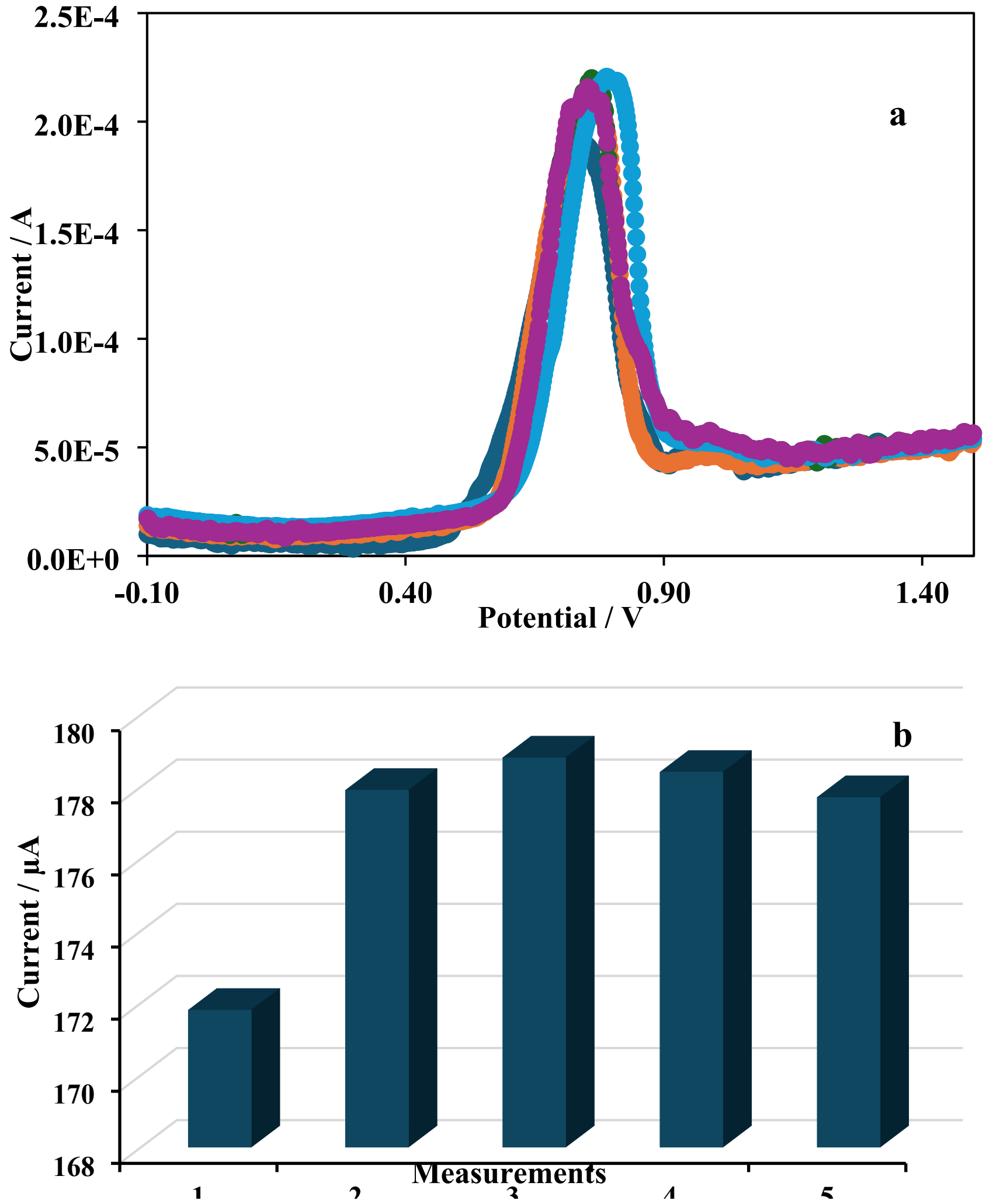

Fig. S5. Repeatability of the MWCNT/GCE modified sensor for 1.0 ppm α-T in 0.1 M $NaClO_4$ prepared in ACN.

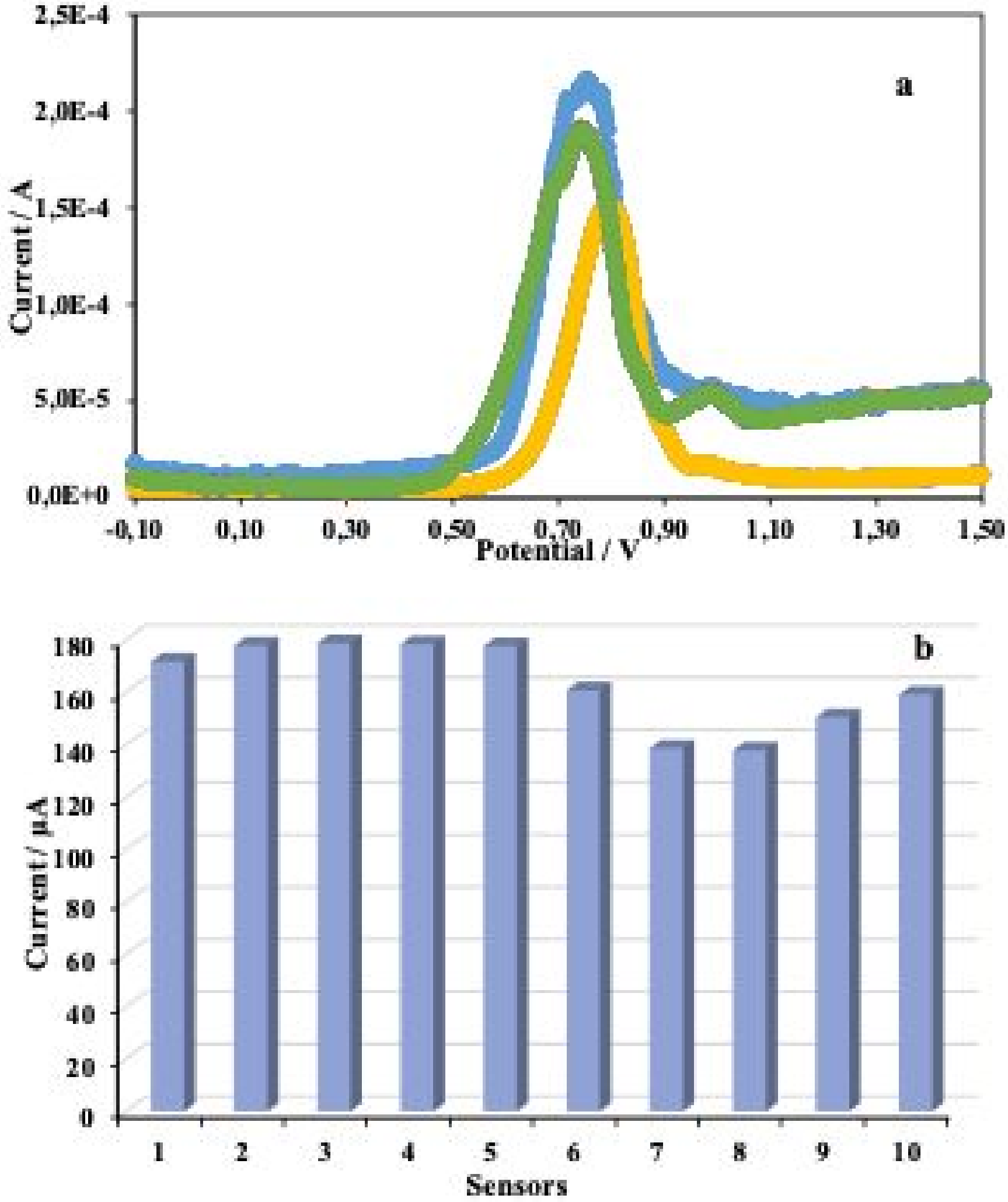

Fig. S6. Reproducibility of MWCNT/GCE modified sensors for 1.0 ppm α-T in 0.1 M NaClO4 prepared in ACN.

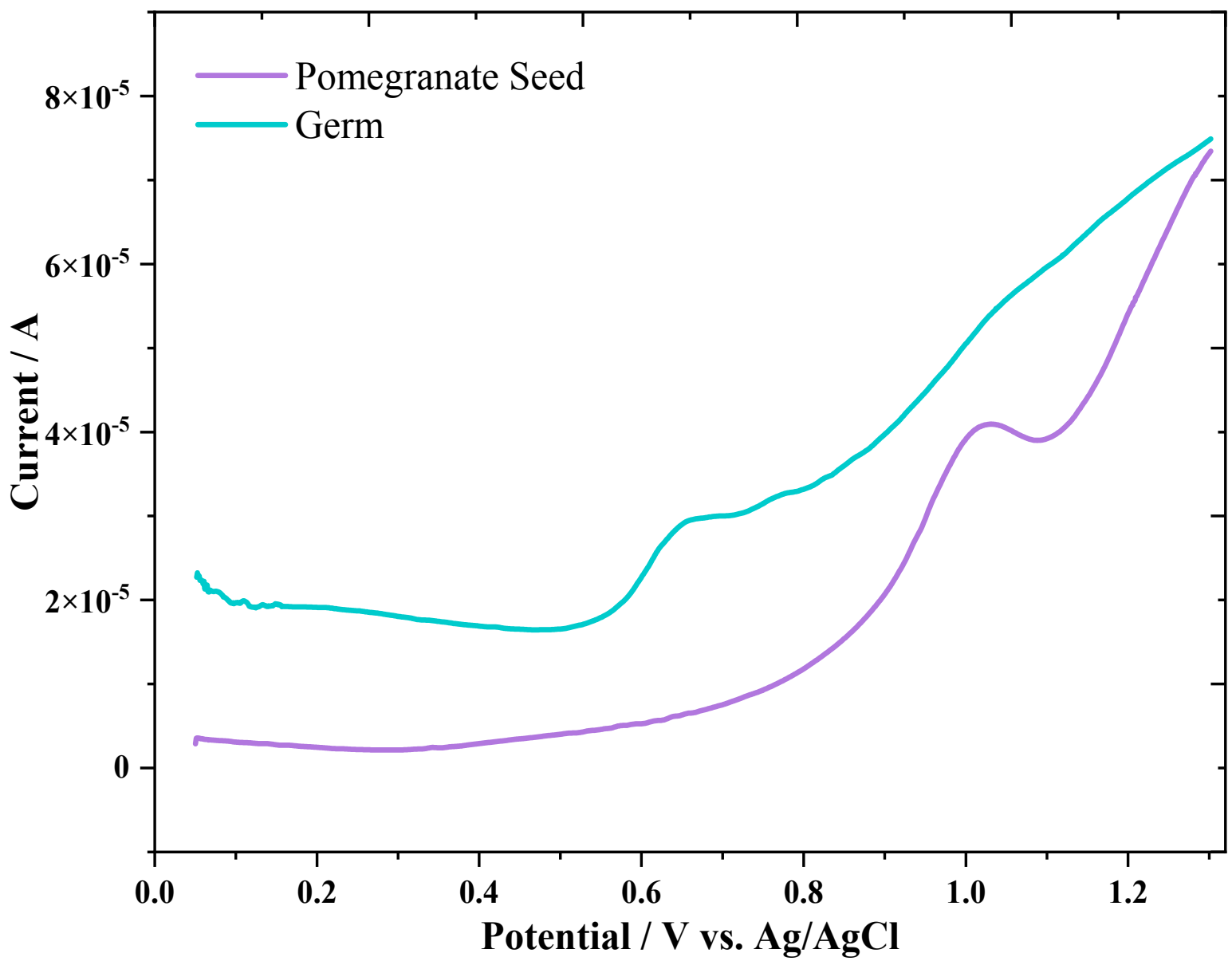

Fig. S7. DPV responses at the MWCNT/GCE modified sensor as a result of diluting the oil samples with 0.1 M $NaClO_4$ in ACN solution.
